# Supplementary material for: Disruption of glycolytic flux is a signal for inflammasome signaling and pyroptotic cell death
Source: eLife. 2016 Mar 24;5:e13663. doi: 10.7554/eLife.13663 (PMC4846378; doi:10.7554/eLife.13663)

**Supplemental File 4: Compound Characterization and Spectra**

az-GB (**6**) – HPLC/MS Trace

az-GB (**6**) - ^1^H-NMR
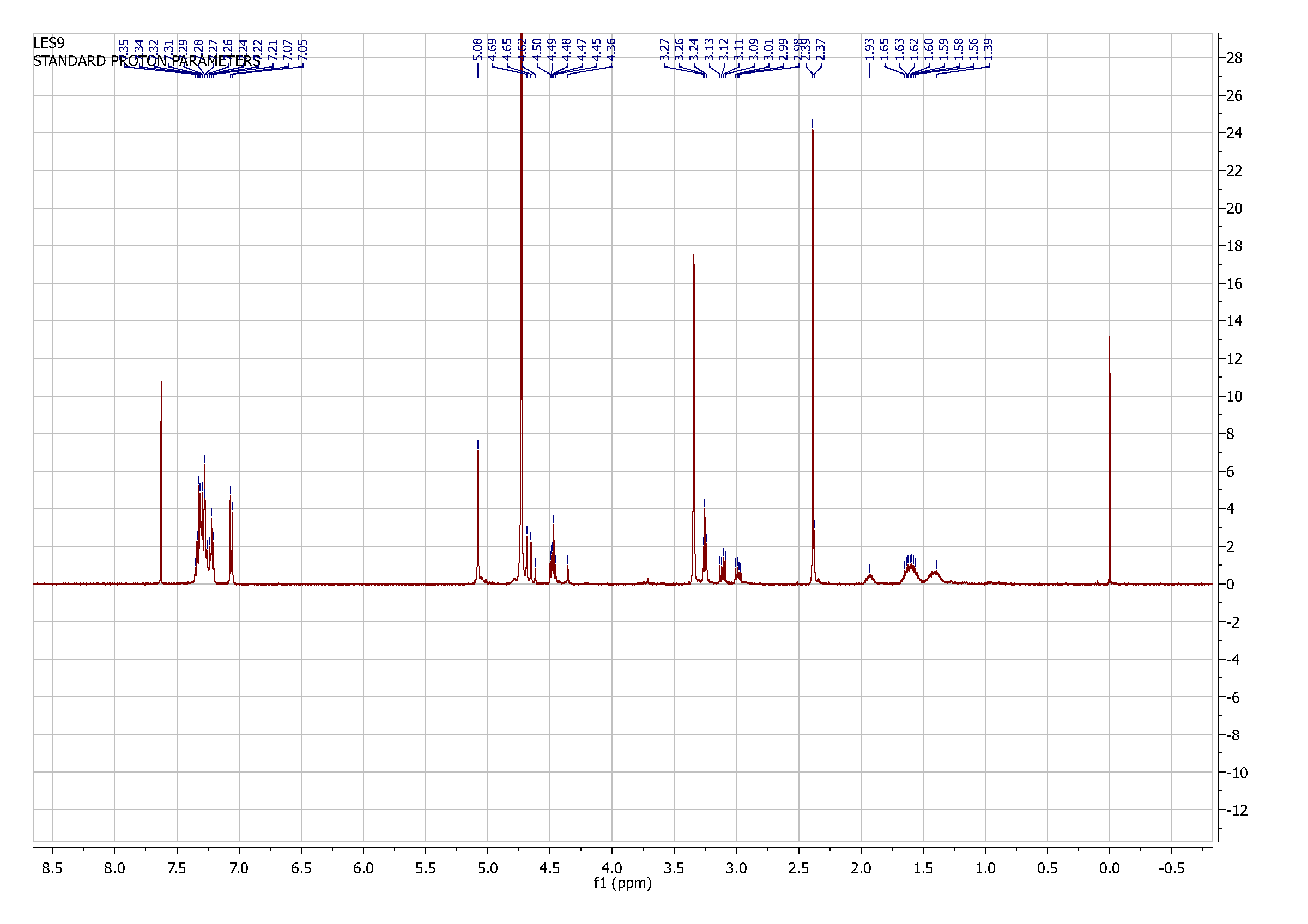


GB111-PMK (**2**) – HPLC/MS Trace

GB111-PMK (**2**) – ^1^H-NMR Spectrum


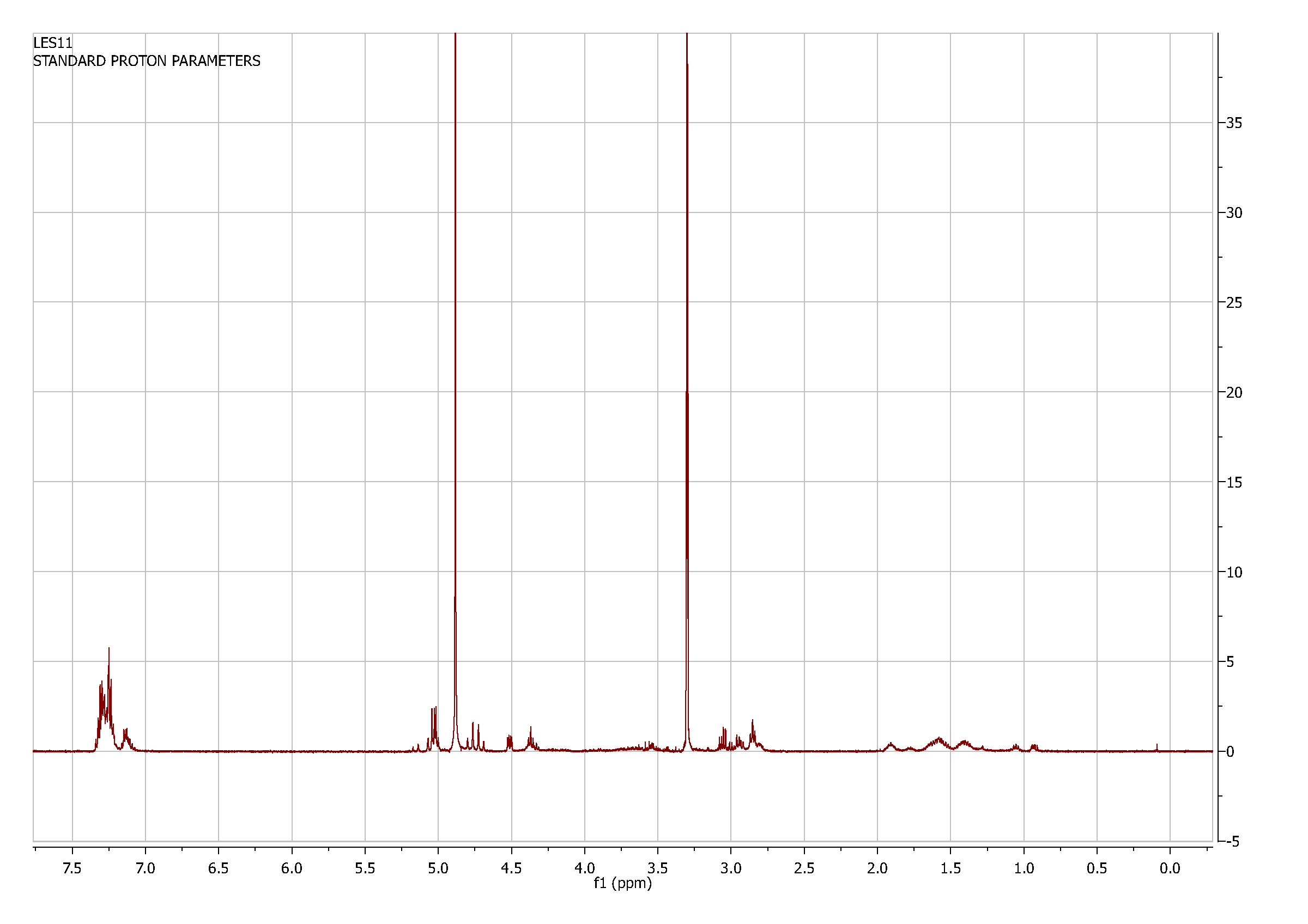


NR-GB111 (**3**) – HPLC/MS Trace

NR-GB111 (**3**) – ^1^H-NMR Spectra


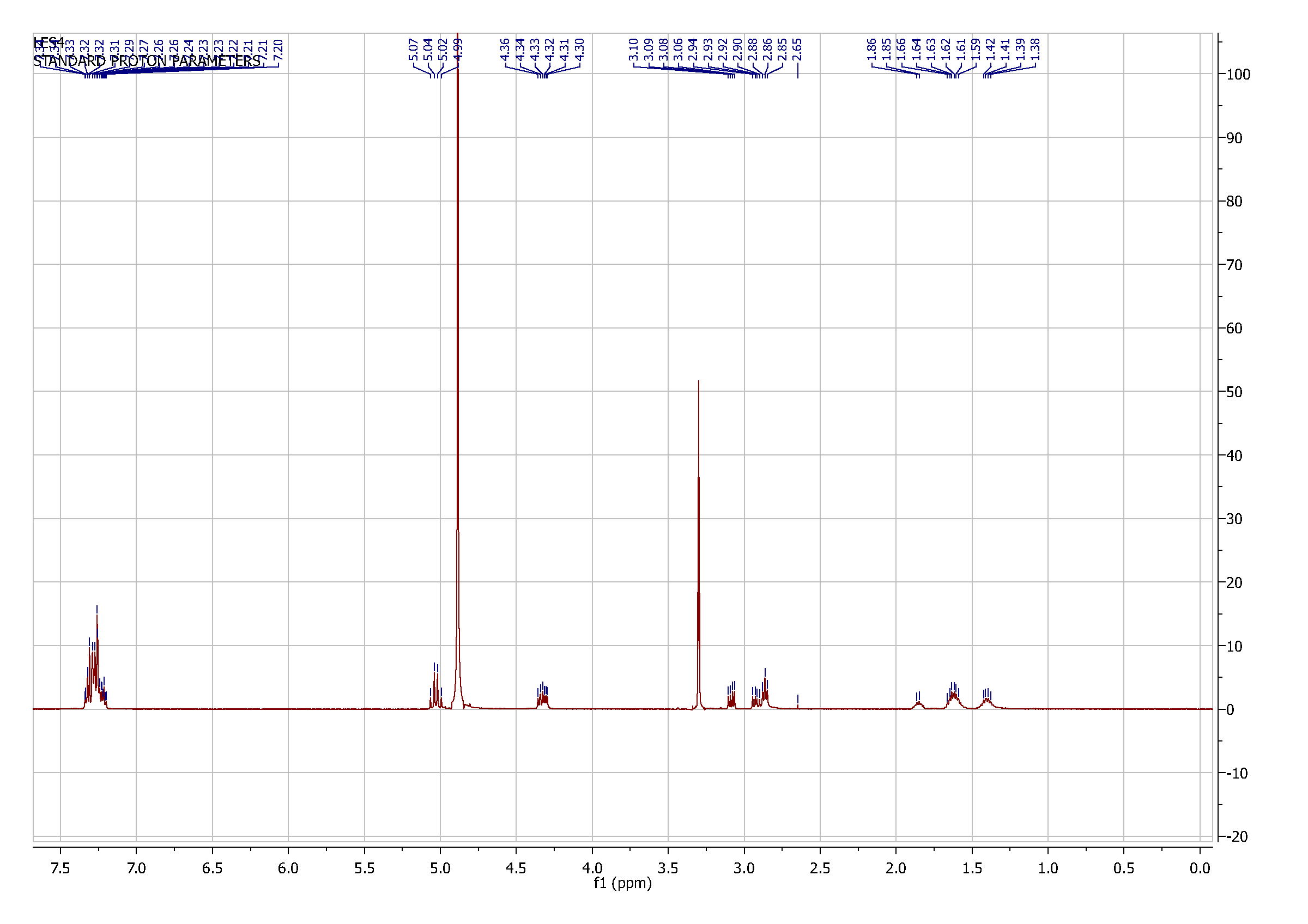


GB-IA (**4**) –HPLC/MS Trace

GB-IA (**4**) – ^1^H-NMR
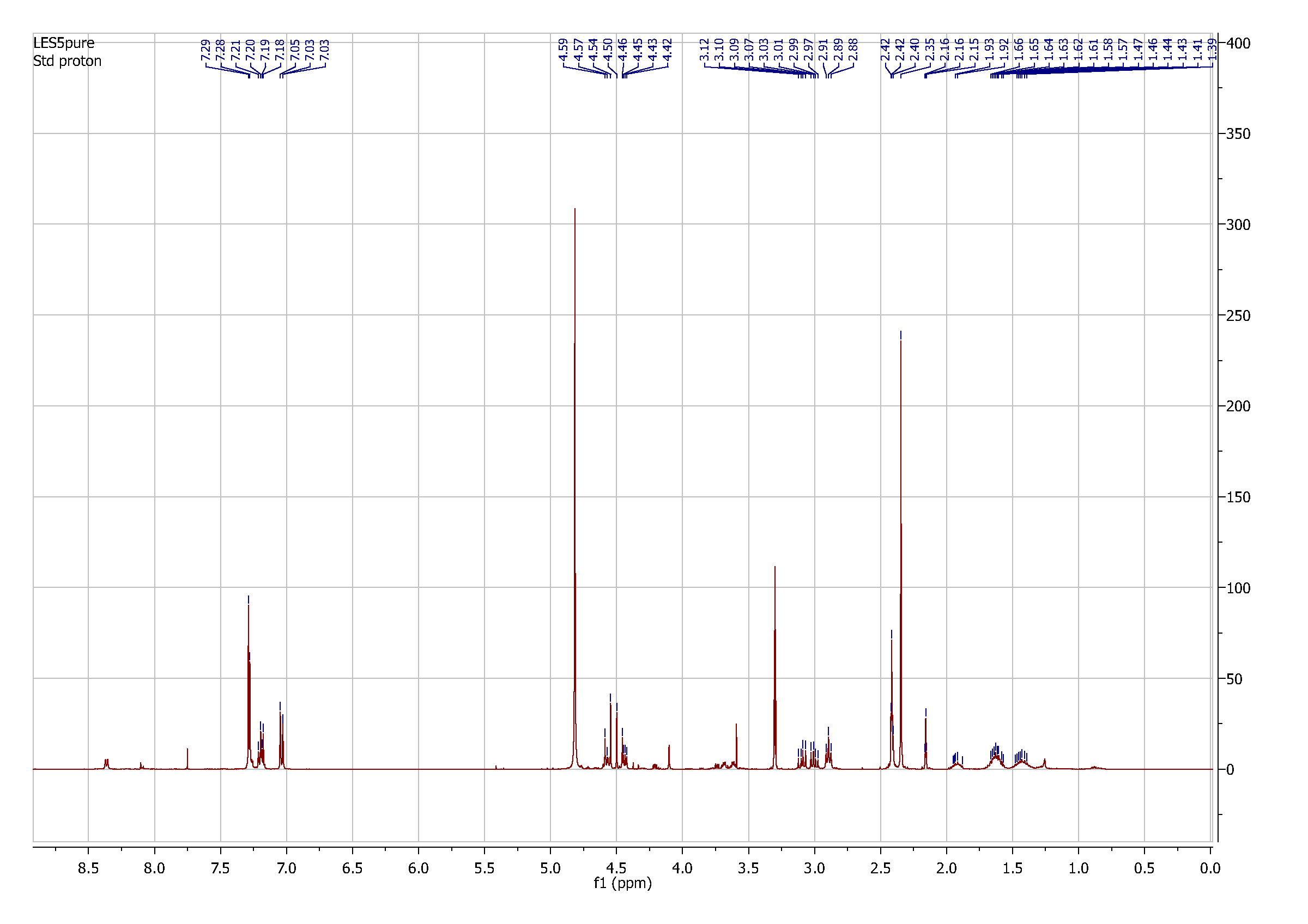


ac-GB111 (**5**) – HPLC/MS Trace

ac-GB111 (**5**) – ^1^H-NMR Spectrum


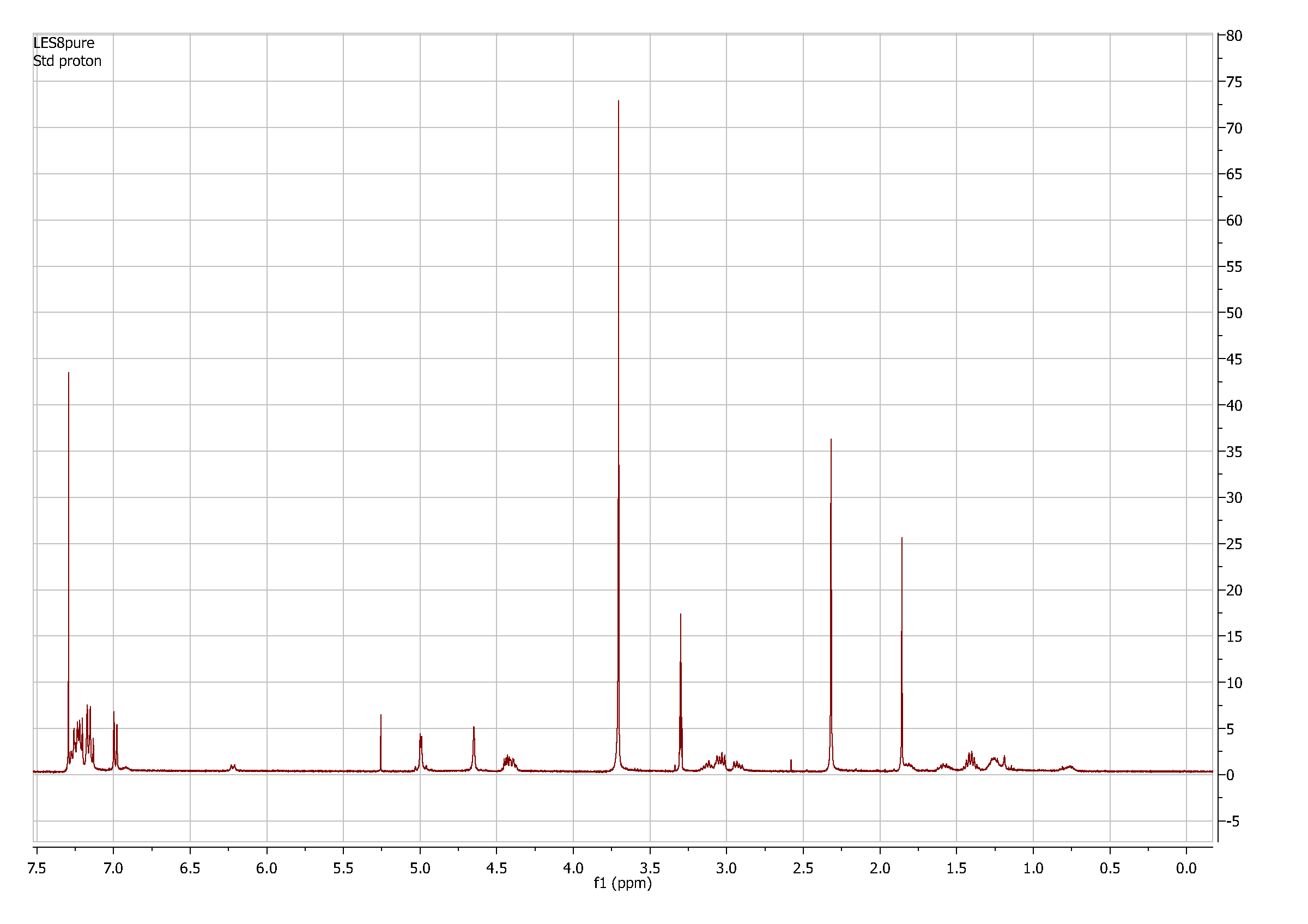

Supplement: Supplementary file 4. — DOI: http://dx.doi.org/10.7554/eLife.13663.038 [file elife-13663-supp4.docx]
